# Efficacy of Platelet-Rich Plasma on Pain and Function in the Treatment of Knee Osteoarthritis: A Prospective Cohort Study

**DOI:** 10.7759/cureus.13909

**Published:** 2021-03-15

**Authors:** Adel H Hegaze, Amre S Hamdi, Abdulraof Alqrache, Mohamed Hegazy

**Affiliations:** 1 Department of Orthopedics, Faculty of Medicine, King Abdulaziz University, Jeddah, SAU; 2 Clinical Biochemistry, King Abdulaziz University, Rabigh, SAU; 3 Trauma and Orthopedics, Independent Researcher, Private Sector, Heidelberg, DEU

**Keywords:** knee osteoarthritis, platelet rich plasma, nrs pain score, intra-articular injection, knee pain

## Abstract

Background

Osteoarthritis (OA) is a degenerative disease commonly affecting the knee joints. It affects patients socially, psychologically and economically and rates of the disease have been increasing due to obesity and old age. Regardless of choosing a medically conservative approach, it is a challenge in the long term to provide OA patients efficient treatment with minimal side effects and long-term efficiency. Platelet-rich plasma (PRP) is a convenient, low-cost and affordable treatment technique used in treating knee OA with encouraging efficient and safe outcomes. In this study we will investigate the effect of PRP on knee OA.

Methods

This is a prospective cohort study involving 252 patients with different OA grades. The Kellgren and Lawrence (K&L) system was used in classifying the affected knee by degenerative cartilage lesions as well as early and severe OA. All patients with a diagnosis of knee OA were screened in every visit before the injection, the pain was assessed by the 0-10 Numeric Rating Scale (NRS), and knee range of motion including flexion and extension was assessed by goniometer. Follow-up appointments were done on three-month intervals for a total of three visits for evaluation. Injection of PRP was given to all the patients with a maximum of four injections. The results were evaluated statistically according to the total number of follow-up visits.

Results

In grade II patients, the pain improved with the visits and the maximum improvement in flexion degree was noticed in patients who came for a total of three follow-up visits. In grade III patients, the most improvement in pain was in patients who came for three follow-up visits, while the most improvement in flexion degree was in patients who came for a total of two follow-up visits. Patients with grade IV who came for three follow-up visits showed the most improvement in pain and degree of flexion.

Conclusions

Intra-articular injections gave significant pain and flexion improvement in grades II, III and IV in OA patients, especially with multiple injection in the short-term follow-up. As a result, recommendation of repeated multiple injections up to four times is efficient in providing long time relief in knee OA.

## Introduction

Osteoarthritis (OA) is the most common type of arthritis and degenerative disease affecting around 250 million globally [1]. It affects patients socially, psychologically and economically and rates of the disease have been increasing due to obesity and old age [1-3]. In the United States, the estimated prevalence of OA is 12% compared to 8.1% in China, accounting for around 110 million OA patients globally [1-4]. Moreover, those under the age of 50 have a prevalence rate of 5.2% while individuals aged 60 are near 11% [4]. In addition to aging and obesity, several risk factors for OA include gender, genetics, smoking and previous joint injury [1]. Moreover, other illnesses such as rheumatoid arthritis and metabolic disorders can decrease bone density, increasing the susceptibility to develop OA [3]. OA patients' common complaints are usually pain, swelling, limitation of movement and stiffness [3]. Such symptoms greatly affect the joint stiffness leading to a decreased range of motion leading to impairment of daily life activities [2,4]. From the time of Hippocrates until 250 years ago, it was believed that the cause of joint pain was gout [5,6]. In 1957, Kellgren and Lawrence reported one of the first radiographic classification and scoring systems of OA, grading it from 0 to 4 according to diminished joint space, loss of cartilage, presence of cysts, sclerosis and osteophytes [1,7]. However, a combined clinical approach with history, examination and radiological findings are still utilized collectively to be able to diagnose OA [1,3]. The most common symptoms found with OA are joint pain, stiffness, swelling and decreased range of motion with the progression of OA being related to the progression of worsening symptoms [1,6].

The articular cartilage is composed of hyaline cartilage, which protects the subchondral bone, redistributes the applied loads, acts as a shock absorber and with the presence of synovial fluid reduces the friction inside the joints. Moreover, growth factors and cytokines including insulin-like growth factor 1 (IGF1), fibroblast growth factor (FGF) and transforming growth factor-beta (TGF-ϐ) play a critical role in the control of chondrogenesis, directing the differentiation of mesenchymal stem cells into mature chondrocytes [7]. Under normal conditions, mesenchymal stem cells form and secrete chondroblasts, macromolecules and collagens (primarily collagen type II), and proteoglycans. Collagen is usually found associated and crosslinked with proteoglycans, forming the structural unit of the extracellular matrix (ECM). Aggrecan is the most common and largest proteoglycan found within the cartilage providing its tensile strength. In addition, proteoglycans can be seen attached to glycosaminoglycans (GAGs) and in return is attached to the aggrecan link protein which consists of chondroitin sulfate and keratin sulfate.

Platelet-rich plasma (PRP) is a convenient, affordable treatment technique used in treating knee OA with encouraging efficient and safe outcomes. Furthermore, it is capable of producing a biological process that gives ambitious results in treating patients with OA [8]. The autologous blood driven from PRP contains a high concentration of platelets. When activated, it releases growth factors, transforming growth factor-β, IGF1 and vascular endothelial growth factor which can have great healing and remodeling local effect on the joint cartilage [9]. In this study we aim to assess the effect of the autologous PRP intraarticular injection in improving knee OA.

## Materials and methods

**Figure 1:**
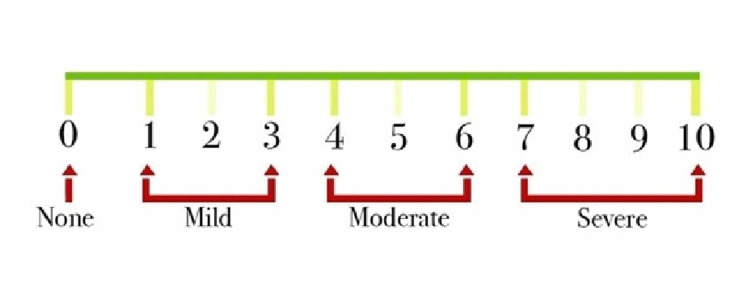
recorded pain score using numerical rating scale

This is a prospective cohort study that was done by the orthopedic outpatient clinic at King Abdulaziz University in Jeddah, Saudi Arabia between June 2018 to June 2019 and was approved by the Institutional Review Board (IRB) of King Abdulaziz University. We included patients who had a confirmed diagnosis of OA with presence of osteoarthritic changes in their radiographic imaging. Meanwhile, we excluded all the patients who had grade I OA, a single PRP injection only, previous intraarticular fracture, previous septic arthritis, previous intra-articular injection of corticosteroids, known cases of rheumatoid arthritis and known cases of anemias. A total of 252 patients with either unilateral or bilateral knee OA with a total of 260 knees were included in the study. All patients had complete blood count (CBC) and a weight-bearing view x-ray done. The grade of OA was determined according to the Kellgren-Lawrence (KL) grading system [10]. Consent was obtained from all the participants before every PRP injection session. Patients were treated by three successive intraarticular PRP injections with one-month intervals giving follow-up appointments after three, six and nine months for evaluation. Some patients missed the third injection and a limited number needed a further fourth injection to evaluate the maximum number of possible PRP injections. In the first visit before the injection, we recorded the height and weight to calculate the body mass index (BMI) and recorded pain score using a numerical rating scale, where 0 = no pain, 1-3 = mild pain, 4-6 = moderate pain, and 7-10 = severe pain (Figure 1).

**Figure 2:**
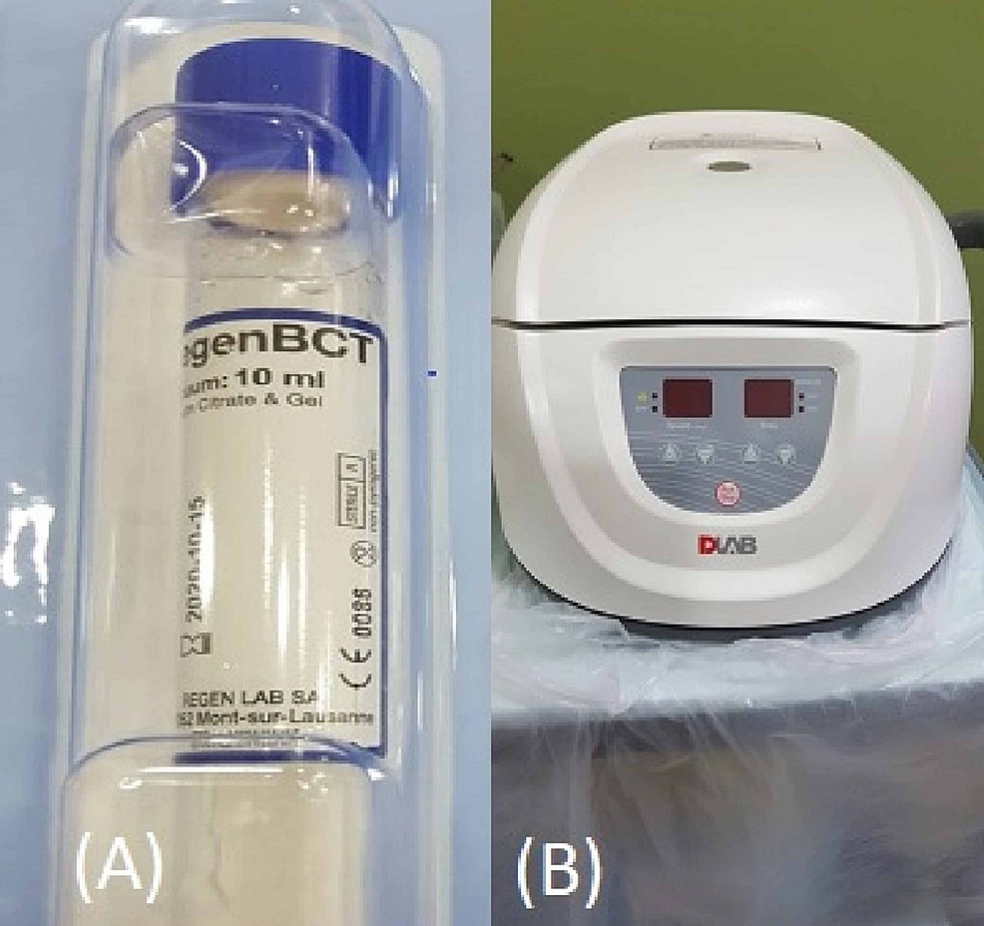
A) Regen Lab SA tube. B) Centrifuge machine used at a speed of 36,000 rounds/min for 6 minutes

The range of movement as flexion and extension was measured using a goniometer. A Phlebotomy technician withdrew 7ml blood from the patients for each joint using a Regen Lab SA (Mont-sur-Lausanne, Switzerland) tube and centrifuged it at a speed of 36,000 rounds/min for six minutes. Afterward, separated plasma was obtained and injected immediately in the prepared knee under a completely aseptic environment (Figure 2).

**Figure 3:**
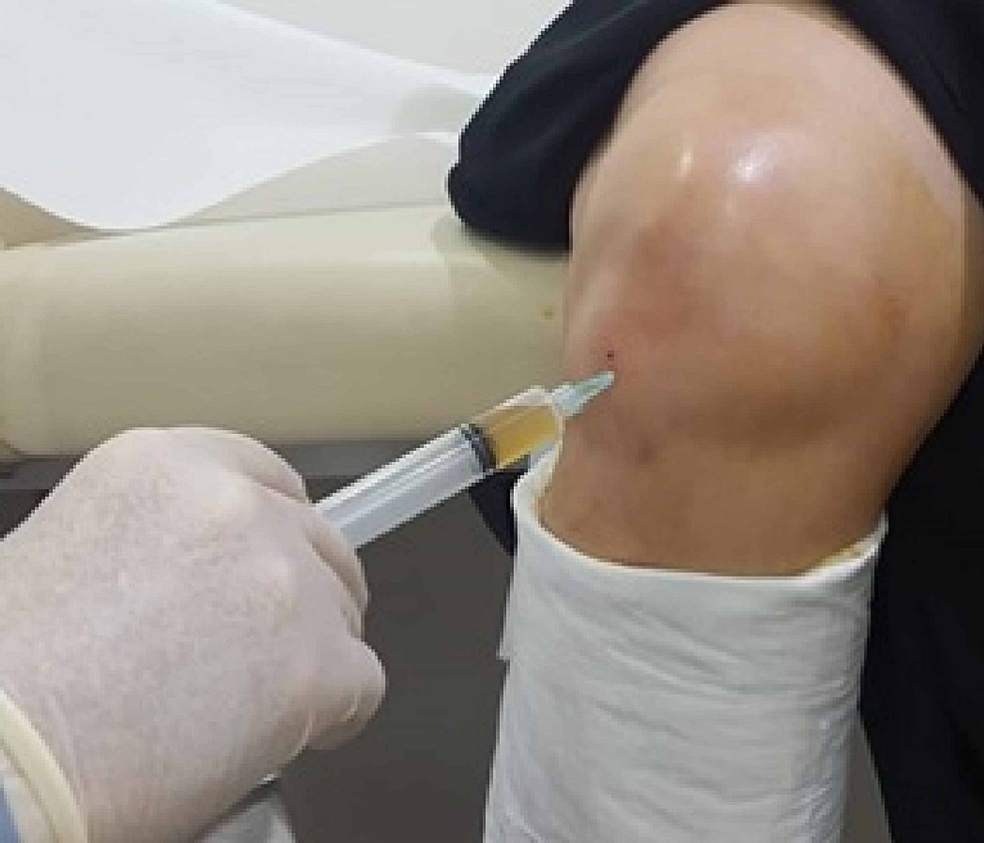
Injection of 5cc of Platelet Rich Plasma to the affected knees 1 cm lateral to patellar tendon and 1 cm below the joint line

We injected 5cc of PRP to the affected knees 1 cm lateral to the patellar tendon and 1 cm below the joint line (Figure 3). Finally, we used Statistical Package for Social Sciences (SPSS) version 26 (IBM Corp., Armonk, NY, USA) for data analysis. An analysis with Wilcoxon signed-rank tests between consecutive visits was conducted, with p-value ≤0.05 considered statistically significant.

## Results

In our study, a total of 260 knees with osteoarthritis ranging between grades 2 to 4 were treated with PRP injections. The average age of participants was 61.46 ± 8.94 and most of them were females (76.9%). Furthermore, both knee sides were almost evenly distributed with the left knee (132; 50.8%) slightly more than the right knee (128; 49.2%). Regarding BMI, around one-third of the patients were overweight (81; 32.3%) and around one-third were class I obese (85; 33.8%). Out of the 260 osteoarthritic knees included, 68 (26.2%) were grade II OA, 112 (43.1%) were grade III OA and 80 (30.8%%) were grade IV OA. Moreover, a total of 124 (47.7%) knees had a total of two PRP injections, 92 (35.4%) had a total of three, and only 44 (16.9%) needed a total of four injections (all grades) with the mean average number of visits 2.69 ± 0.74.

For patients with grade II OA who received two PRP injections (n = 40 knees), there was a marked drop in pain from an average of 6 to 3.8 between the initial visit and the second follow-up and was statistically significant (P-value = 0.000). However, there was no remarkable decrease in the degree of flexion as it decreased from 122.10^o^ to 121.90^o^ (P-value = 0.652). Meanwhile, in patients who received three PRP injections (n = 12 knees), the pain was reduced from an average of 6.67 in the initial visit (P-value = 0.087) to 5 in the first follow-up but slightly increased to an average of 5.67 in the second follow-up and was statistically significant (P-value = 0.005). Regarding the change in the degree of flexion, a small increase was noted from 122.33^o^ in the initial visit to 123.33^o^ in the first follow-up (P-value = 0.01) and was further reduced in the second follow-up to 114^o^ (P-value = 0.812). Lastly, patients who received four PRP injections (n = 16 knees) showed no significant change in pain between the initial visit and the first follow-up, however, a change was noticed in the second follow-up as the pain decreased from an average of 6.25 in the first follow-up to a 4.75 in the second visit and substantial decrease to an average of 2.25 in the third visit. Interestingly, they were found to be statistically significant during the second and third follow-up (P-value = 0.000, P-value = 0.002, respectively). Similarly, for changes in the degree of flexion, there was a small increase from 120.25^o^ in the initial visit to 123.75^o^ in the first follow-up, reduced to 118.25^o^ in the second follow-up, and improved to 126.50^o^ in the third follow-up and were significant in both the second and third follow-up (Table 1).

**Table 1:** the difference between visits regarding pain scale and flexion degree in grade II Osteoarthritis patients Z score and P-value calculated using Wilcoxon signed-rank tests between the visit and the one before it. * Statistically significant with P-value ≤ 0.05

Grade II		Initial pain	1^st^ follow up (3 months)	2^nd^ follow up (6 months)	3^rd^ follow up (9 months)	Initial flexion	1^st^ follow up (3 months)	2^nd^ follow up (6 months)	3^rd^ follow up (9 months)
2 Visits	Mean	6	3.8	-	-	122.10^o^	121.90^o^	-	-
	Median	6.5	4	-	-	120^o^	125^o^	-	-
	Z score	-	-5.607	-	-	-	-0.451	-	-
	P-value	-	0.000*	-	-	-	0.652	-	-
3 Visits	Mean	6.67	5	5.67	-	122.33^o^	123.33^o^	114^o^	-
	Median	8	5	6	-	129^o^	130^o^	116^o^	-
	Z score	-	-1.71	-2.828	-	-	-2.828	-0.238	-
	P-value	-	0.087	0.005*	-	-	0.01*	0.812	-
4 Visits	Mean	6	6.25	4.75	2.25	120.25^o^	123.75^o^	118.25^o^	126.50^o^
	Median	6	6	4.5	2	122.50^o^	130^o^	120^o^	125^o^
	Z score	-	-0.545	-3.704	-3.095	-	-1.789	-3.053	-3.159
	P-value	-	0.586	0.000*	0.002*	-	0.074	0.002*	0.002*

For patients with grade III OA who received two PRP injections (n = 52 knees), there was a significant reduction in pain from 7 in the initial visit to 5.85 in the follow-up (P-value = 0.007), and a small increase in the degree of flexion from 110.31^o^ to 113.23^o^ (P-value = 0.044). For patients who received three PRP injections (n = 48 knees), there was a decrease in pain from 6.67 in the initial visit to 4.08 in the first follow-up (P-value = 0.000), followed by a non-significant increase in the second follow-up (P-value = 0.867). Regarding the change in the degree of flexion, there was a gradual increase in the degree from 114.33^o^ in the initial visit to 115.33^o^ in the first follow-up (P-value = 0.231) and a significant increase of 121.42^o^ in the second follow-up (P-value = 0.000). Lastly, for patients who received four PRP injections (n = 12 knees), there was a significant improvement of pain throughout three follow-ups as well as in degree of flexion (P-value ≤ 0.01). The pain started at 7.67 and decreased to 6.33 in the first follow-up, decreased further to 5.33 in the second visit, and decreased substantially to an average of 3.33 in the third one. In the same context, the degree of flexion increased from 123.33^o^ to 124^o^ in the second follow-up, then to 127^o^ in the third visit (Table 2).

**Table 2:** the difference between visits regarding pain scale and flexion degree in grade III osteoarthritis patients Z score and P-value calculated using Wilcoxon signed-rank tests between the visit and the one before it. * Statistically significant with P-value ≤ 0.05

Grade III		Initial pain	1^st^ follow up (3 months)	2^nd^ follow up (6 months)	3^rd^ follow up (9 months)	Initial flexion	1^st^ follow up (3 months)	2^nd^ follow up (6 months)	3^rd^ follow up (9 months)
2 Visits	Mean	7	5.85	-	-	110.31^o^	113.23^o^	-	-
	Median	6	6	-	-	110^o^	110^o^	-	-
	Z score	-	-2.712	-	-	-	-2.012	-	-
	P-value	-	0.007*	-	-	-	0.044*	-	-
3 Visits	Mean	6.67	4.08	4.25	-	114.33^o^	115.33^o^	121.42^o^	-
	Median	6	4	4	-	112.50^o^	117^o^	121^o^	-
	Z score	-	-6.1	-0.167	-	-	-1.198	-4.095	-
	P-value	-	0.000*	0.867	-	-	0.231	0.000*	-
4 Visits	Mean	7.67	6.33	5.33	3.33	123.33^o^	124.33^o^	124^o^	127^o^
	Median	8	6	6	3	130^o^	130^o^	128^o^	130^o^
	Z score	-	-3.176	-2.585	-2.585	-	-2	-1.149	-2.585
	P-value	-	0.001*	0.01*	0.01*	-	0.046*	0.251	0.01*

**Figure 4:**
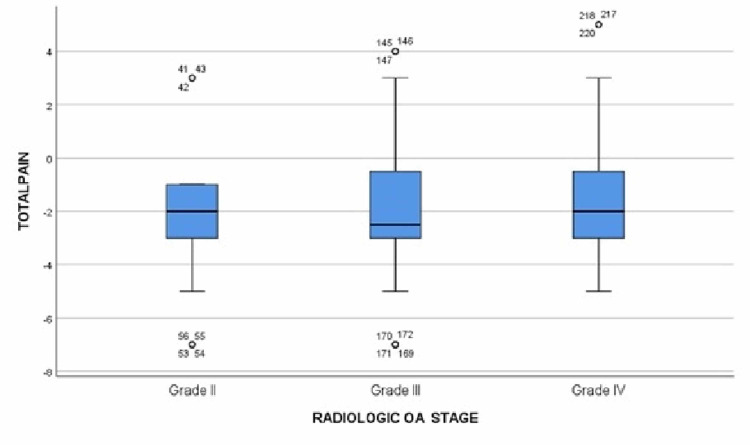
Boxplot showing the relation between pain changes and osteoarthritis grades OA: osteoarthritis

**Figure 5:**
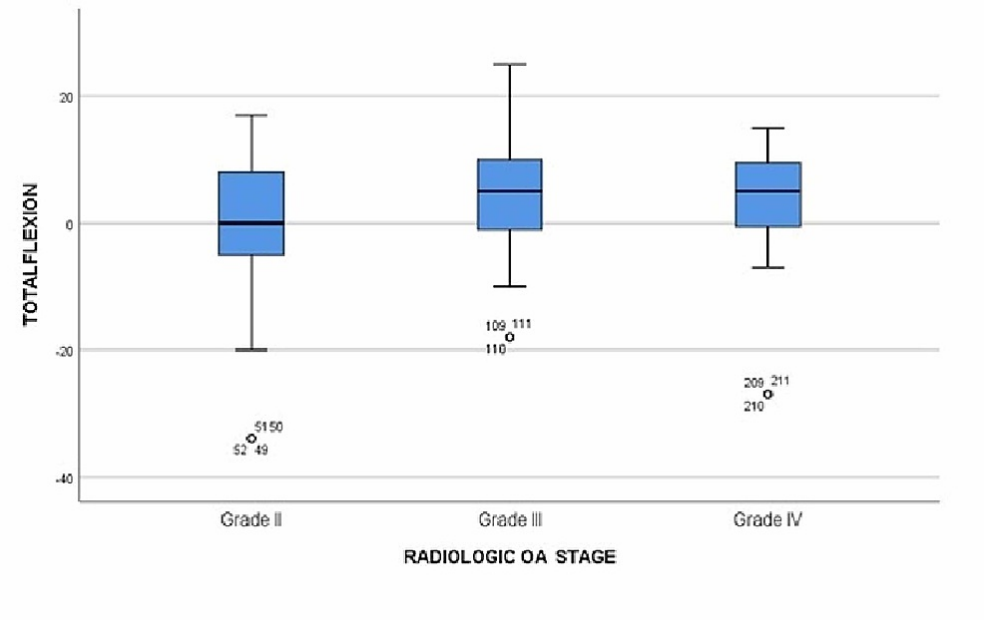
Boxplot showing the relation between changes in flexion degree and osteoarthritis grades OA: osteoarthritis

For patients with grade IV OA who received two PRP injections (n = 32 knees), there was an improvement in pain from 7.62 in the initial visit to 5.25 in the follow-up (P-value = 0.000) and the degree of flexion increased from 111.63^o^ in the initial appointment to 112.38^o^ in the follow-up (P-value = 0.0146). For patients who received three PRP injections (n = 32 knees), the average pain score was at a plateau level of 5.8 in all visits with no significant changes. However, regarding the difference in the degree of flexion, there was no significant change between the initial visit and the first follow-up appointment but there was an increase from 105.63^o^ in the first visit to 109^o^ in the second visit (P-value = 0.001). For patients receiving four PRP injections (n = 16 knees), there was a gradual decrease in pain score from 8.5 in the initial visit to an average of 6.5 in the first follow-up (P-value = 0.000) and a further decrease of average score of 4.25 in the second follow-up (P-value = 0.000), followed by a slight increase to 5.25 in the third follow-up visit (P-value = 0.087). For the change in the degree of flexion, there was a significant gradual improvement from 108.75^o^ in the initial visit to 115^o^ in the first follow-up (P-value = 0.000) but no significance was found in the second follow-up where the average flexion degree was recorded at 117.25^o^, then decreased to 114.25^o^ in the third follow-up visit (Table 3). The overall changes in pain and flexion are mentioned in Table 4 and plotted on boxplots in Figure 4 and Figure 5. A Spearman's test correlation was used to find the relation between variables. Strong negative relationships were found between overall change in pain and the total number of visits (R = 0.185, P-value = 0.003), and a positive correlation between the overall changes in the degree of flexion and the total number of visits (R = 0.139, P-value = 0.025).

**Table 3:** the difference between visits regarding pain scale and flexion degree in grade IV osteoarthritis patients Z score and P-value calculated using Wilcoxon signed-rank tests between the visit and the one before it. * Statistically significant with P-value ≤ 0.05

Grade IV		Initial pain	1^st^ follow up (3 months)	2^nd^ follow up (6 months)	3^rd^ follow up (9 months)	Initial flexion	1^st^ follow up (3 months)	2^nd^ follow up (6 months)	3^rd^ follow up (9 months)
2 Visits	Mean	7.62	5.25	-	-	111.63^o^	112.38^o^	-	-
	Median	8	5	-	-	111.50^o^	112^o^	-	-
	Z score	-	-4.681	-	-	-	-1.997	-	-
	P-value	-	0.000*	-	-	-	0.046*	-	-
3 Visits	Mean	5.63	5.88	5.88	-	105.25^o^	105.63^o^	109^o^	-
	Median	6	6	5	-	107.50^o^	110^o^	107.50^o^	-
	Z score	-	-0.117	-0.07	-	-	-0.466	-3.289	-
	P-value	-	0.907	0.944	-	-	0.641	0.001*	-
4 Visits	Mean	8.5	6.5	4.25	5.25	108.75^o^	115^o^	117.25^o^	114.25^o^
	Median	8	6.5	4	5	110^o^	114.50^o^	119^o^	120^o^
	Z score	-	-3.579	-3.579	-1.71	-	-3.54	-0.947	-0.244
	P-value	-	.000*	.000*	0.087	-	.000*	0.343	0.807

**Table 4:** the overall change in pain (negative values = improvement) and flexion (positive values = improvement)

O.A. Stage	Total Number of Follow Up Visits	Overall Change in Pain	Overall Change in Flexion
Grade II	1	-2.20	-.20^o^
	2	-1.00	-8.33^o^
	3	-3.75	6.25^o^
Grade III	1	-1.15	2.92^o^
	2	-2.42	7.08^o^
	3	-4.33	3.67^o^
Grade IV	1	-2.37	.75^o^
	2	.25	3.75^o^
	3	-3.25	5.50^o^

## Discussion

The knee joint is the most common joint affected by osteoarthritis while total knee arthroplasty (TKA) is usually indicated for late-stage cases not responding to different modalities of conservative methods and medication as a last resort. It is estimated that up to one-third of TKA patients experience chronic pain postoperatively resulting in a reported low outcome rate of 20% [11]. However, the conservative treatment can be met by various difficulties limiting its effect [12]. Although there is no conclusive curative treatment for OA medically, the primary goal of conservative treatment is to relieve pain and improve joint mobility, function and lifestyle [12]. Regarding conservative treatment, it consists of an array of pharmacological drugs including analgesics, such as acetaminophen (paracetamol, oral non-steroidal anti-inflammatory drugs (NSAIDs), topical NSAIDs, physiotherapy and exercise, and intra-articular injection by glucocorticoids and hyaluronic acid (HA), i.e. viscosupplements [13]. However, pharmacological drugs can have adverse side effects with concerns about their safety in long-term administration. On the other hand, non-pharmacological treatment, such as physiotherapy, has limited overall clinical improvements in symptoms. In addition, exercise treatments often have poor outcomes as it is difficult to make the patient commit and comply with a long-term exercise regimen. Meanwhile, intra-articular glucocorticoids offer temporary pain relief for short periods of a few weeks at most [14]. The use of intra-articular injections of viscosupplements is useful as it reduces the use of pharmacologic drugs especially for elderly patients who are often found treated with other comorbidities. The treatment with HA gives an improvement in pain similar to NSAIDs, but it is safer as it is locally injected and requires frequent injection over a regular period of sessions [13].

PRP is an autologous or allogenic blood-derived component composed of concentrated platelet of the blood, which can activate and accelerate repair of the connective tissue. Ever since the first research about PRP, which was done by Sampson et al. [15], there was a considerable number of researches studying the effect of PRP in the treatment of knee OA. In general, results showed that PRP is a safe treatment modality with a financial limited cost and can provide symptomatic and functional improvement in the long term. Furthermore, it is easy to use with minimum skills and reduces the patient’s consumption of oral medicine [12,15,16]. Various studies carried out afterward on intra-articular injection with PRP for the treatment of degenerative knee OA showed pain reduction, improvement in knee function and quality of life [17,18]. Furthermore, various studies were also done comparing the result and efficacy of PRP administration to HA which concluded that autologous PRP injections showed more and longer efficacy than HA injections in reducing pain and symptoms as well as in restoring articular function. Moreover, it is considered more effective and safer in the treatment of the initial stages of knee osteoarthritis than any other treatment approach, making it the most superior conservative treatment to date [19]. Meanwhile, different studies compared between intra-articular injection of PRP, HA, saline and placebo reported that intra-articular PRP injections have more benefit in pain relief and functional improvement in patients with symptomatic knee OA up to one-year post-injection [20,21].

Other studies that were done to investigate the efficacy of a single or multiple PRP injections for knee OA showed that a single injection was as effective as multiple PRP injections in pain improvement. However, multiple injections seemed more effective in joint functionality than a single injection at a six-month interval [18,22]. Recently, research studying the efficacy of PRP in the treatment of knee OA found that PRP is superior to other intraarticular injectable options [23,24]. In addition to the benefits of pain and function improvements, a recent study found that it could prevent disease progression and decrease the likelihood of TKA while showing that it is more cost-effective when compared to HA injections [25,26]. Moreover, Luo et al. studied the effect of PRP injections in overweight and obese patients and concluded that PRP was better in relieving pain and restoring function in that group [27]. In our study, the pain and flexion of the affected knees have improved in almost all the groups. Among all OA grades, the patient who received four PRP injections had the most improvement regarding pain.

In patients with grade II OA knees, an improvement in pain was observed from the first follow-up visit, reaching the maximal improvements in pain and the degree of flexion by the third follow-up visit. The patients who came for a total of three follow-up visits had an improvement in pain by 3.75 points on the NRS and in the degree of flexion by 6.25^o^. Although, in our study, grade II OA knee flexion did not improve except in the patients who received a total of four PRP injections, the overall pain decreased in all grade II groups. This finding is directly in line with Dhillon et al.'s conclusion that PRP injections are an effective treatment for early-stage knee OA symptoms [28].

**Figure 6:**
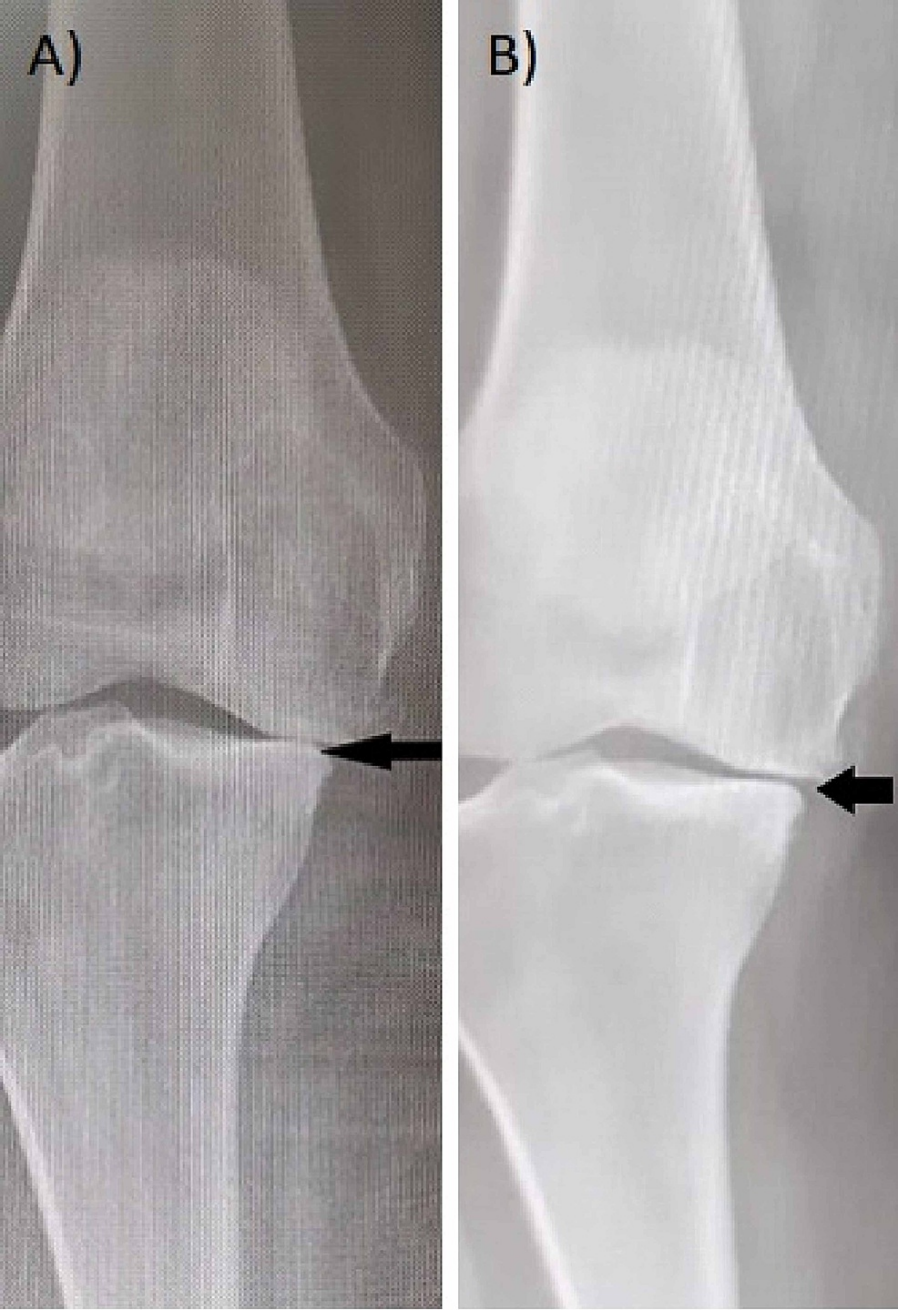
X-rays weight bearing view for the same patient almost one year apart: A) On 16/05/2018 and showing loss of joint space (arrow). B) On 26/04/2019 after treatment of four Platelet Rich Plasma injections showing improvement in joint space (arrow) denote starting cartilage formation.

In a study by Su et al., they excluded grade IV knee OA and stated that intra-articular treatment for grade IV OA will be less effective than both grade II & III [29]. Contrary to their hypothesis, in our study, intraarticular PRP injections decreased the overall pain in grade IV knee OA as much as it did in grade II and III. For the patients with grade IV OA who followed-up once only, the pain improved by 2.37 points on the NRS while the flexion improved by 0.75^o^. However, the pain did not improve in patients who followed-up twice and worsen by 0.25 points on the NRS while the flexion improved by 3.75^o^. A total of three follow-up visits showed an improvement in pain by 3.25 points and in the degree of flexion by 5.50^o^. The flexion range of motion became better with the increase in the total number of visits among grade IV knees, and we observed an overall improvement in flexion in grade IV more than grade II knees. Radiological imaging showed an improvement in our patients in comparison to the patients included in the Raeissadat et al. study (Figure 6) [30].

Despite the positive outcomes that are reported in the literature, the diversity of the methodological studies that include preparation, administration and intervals of the injection. Furthermore, details on storage, centrifugation speed and time for plasma isolation can be variable and have an unnoticed effect on the process. Moreover, the cost of treatment was not explored in our study with having up to four injections. However, we followed the most common interval used of four weeks but a fourth injection was added to explore the effectiveness of the treatment.

## Conclusions

In this study we demonstrated that intra-articular injection of PRP in OA knee in grades II, III and IV are valuable, efficient and cost-effective. Patients observed a decrease in pain and an increase in degree of flexion. The changes were noticeable short term after three, six and nine months of follow-ups. Grade IV gave a good response to injection with improvement in pain and range of flexion, which as a result decreased the possibility of needing TKA or delayed it. Multiple injections gave better results than single injection in all grades regardless of their weight.
